# Association of sex hormone-binding globulin and dyslipidemia with Japanese postmenopausal women: a cross-sectional study

**DOI:** 10.1186/s12944-025-02634-2

**Published:** 2025-06-10

**Authors:** Takahiro Ichikawa, Hiroshi Okada, Masahide Hamaguchi, Megumi Hara, Naoyuki Takashima, Sadao Suzuki, Shiroh Tanoue, Yasufumi Kato, Mako Nagayoshi, Takashi Tamura, Michiaki Fukui, Keitaro Matsuo

**Affiliations:** 1 Department of Endocrinology and Metabolism, Graduate School of Medical Science, Kyoto Prefectural University of Medicine, 465 Kajii-cho, Kawaramachi-Hirokoji, Kamigyo-ku, Kyoto, 602-8566 Japan; 2 Department of Preventive Medicine, Faculty of Medicine, Saga University, 5-1-1 Nabeshima, Saga, 849-8501 Japan; 3 Department of Epidemiology for Community Health and Medicine, Kyoto Prefectural University of Medicine, 465 Kajii-cho, Kamigyo-ku, Kyoto, 602- 8566 Japan; 4 NCD Epidemiology Research Center, Shiga University of Medical Science, Seta-Tsukiwacho, Otsu, 520-2192 Shiga Japan; 5 Department of Public Health, Nagoya City University Graduate School of Medical Sciences, 1 Kawasumi, Mizuho-cho, Mizuho-ku, Nagoya, 467-8601 Japan; 6 Department of Epidemiology and Preventive Medicine, Kagoshima University Graduate School of Medical and Dental Sciences, 8-35-1 Sakuragaoka, Kagoshima, 890-8544 Japan; 7 Department of Preventive Medicine, Nagoya University Graduate School of Medicine, 65 Tsurumai-cho, Showa-ku, Nagoya, 466-8550 Japan; 8 Division of Cancer Epidemiology and Prevention, Aichi Cancer Center, 1-1 Kanokoden, Chikusa-ku, Nagoya, 464-8681 Japan; 9 Department of Cancer Epidemiology, Nagoya University Graduate School of Medicine, 65 Tsurumai-cho, Showa-ku, Nagoya, 466-8550 Japan

**Keywords:** Sex hormone-binding globulin (SHBG), Dyslipidemia, Japanese, Menopause, Cohort

## Abstract

**Background:**

In postmenopausal women, lower levels of sex hormone-binding globulin (SHBG) have been linked to various metabolic conditions. The association between SHBG levels and the presence of dyslipidemia was investigated in comparison with other sex hormones.

**Methods:**

Data from 570 postmenopausal women were analyzed. To assess the relationship between circulating sex hormone concentrations and dyslipidemia, logistic regression and receiver operating characteristic (ROC) curve analyses were performed to assess the relationships.

**Results:**

Participants had a median age of 51.0 years (49.0–53.0). The multivariate analysis revealed that SHBG levels were significantly associated with dyslipidemia. Specifically, low SHBG levels correlated with hypertriglyceridemia and low high-density lipoprotein levels. The area under the curve (AUC) and the optimal SHBG level cutoff value for identifying the presence of dyslipidemia were 0.626 and 69.0 nmol/L, respectively. The AUCs for SHBG levels were highest for estradiol (E2), total testosterone (TT) and dehydroepiandrosterone sulfate (DHEAS) levels.

**Conclusions:**

SHBG levels were significantly associated with dyslipidemia in postmenopausal women and outperformed E2, TT, and DHEAS levels. These findings highlight SHBG as a potential biomarker for dyslipidemia risk in postmenopausal women, warranting further research into its prognostic utility.

**Supplementary Information:**

The online version contains supplementary material available at 10.1186/s12944-025-02634-2.

## Introduction

The global prevalence of dyslipidemia has increased over the past 30 years and has become a significant public health concern worldwide [1]. Dyslipidemia is typically defined by increased levels of total cholesterol, low-density lipoprotein (LDL) cholesterol, and triglycerides, along with decreased levels of high-density lipoprotein (HDL) cholesterol, and is recognized as a primary risk factor for cardiovascular disease [2]. 

The menopausal transition leads to increased LDL cholesterol and apolipoprotein B levels and vascular remodeling in women [3]. Menopause is characterized by changes in serum steroid concentrations including a significant decrease in estradiol (E2) levels and a slight decrease in testosterone levels [4]. In contrast, Pasquali et al. [5]. revealed no significant difference in sex hormone-binding globulin (SHBG) levels before and after menopause. While follicle-stimulating hormone (FSH) is essential for understanding the hormonal milieu in postmenopausal women [6], circulating SHBG, positively correlating with FSH [7], has been consistently associated with various metabolic abnormalities in the same group [8, 9]. SHBG is predominantly synthesized in the liver and is the binding protein for estradiol and testosterone, which regulate biological functions [10]. Although SHBG levels remain stable across menopausal transitions [5], numerous studies have indicated that SHBG levels are linked to metabolic disturbances and cardiovascular conditions in postmenopausal women [8, 9, 11, 12]. Although an association between SHBG levels and dyslipidemia in postmenopausal women has been previously reported [11, 13–15], it remains unclear whether SHBG levels are more strongly associated with dyslipidemia than the other sex hormones.

This study hypothesized that SHBG would show the strongest association with dyslipidemia among postmenopausal women, relative to other circulating sex hormones. To address this, SHBG, E2, total testosterone (TT), and dehydroepiandrosterone sulfate (DHEAS) were comparatively evaluated, providing novel insights into the relative hormonal contributions to lipid metabolism in this population.

## Methods

### Study design

The Japan Multi-Institutional Collaborative Cohort Study (J-MICC), launched in 2005, investigates genetic and environmental factors in lifestyle-related diseases, with a focus on cancer. A detailed description of this cohort study has been published previously [16, 17], and is also provided in Supplementary Table 1. This cross-sectional study used the baseline data from the J-MICC study included naturally menopaused women. The research protocol was reviewed and approved by the ethics committees of all participating institutions. Written informed consent was obtained from all individuals prior to participation. Further details are provided in Supplementary Table 1.

### Participants

In this study, the participants were postmenopausal women with available serum hormone data in the J-MICC study. Natural menopause was defined as the cessation of menstruation for at least 12 consecutive months unrelated to surgery or other obvious causes [18]. 

Participants were excluded if baseline data were missing including lipid, fasting status of blood samples, and age data, or if they had been diagnosed with premature ovarian insufficiency, or using hormone therapy at enrollment.

### Data collection and analytical approaches

Participants were asked to complete a questionnaire on lifestyle and medical conditions and provide a blood sample. The participants completed a self-administered questionnaire that included questions on anthropometric factors and lifestyle. Additionally, the participants were queried regarding their age at menopause onset and the underlying cause. The participants were also asked about hormone therapy history including details on the duration of use and age at initiation and cessation of therapy.

Body mass index (BMI) was calculated by dividing self-reported weight (kg) by the square of height (m²) and was used as a proxy measure of adiposity. Smoking habits were categorized into three groups: individuals who have never smoked, those with a history of smoking, and current smokers. “Not current drinker” was defined as a daily alcohol intake of less than 20 g/day, based on prior research indicating that even low alcohol consumption may increase the risk of cardiovascular disease in Asian populations [19]. Physical activity was evaluated using a format based on the International Physical Activity Questionnaires [20]. It was quantified in metabolic equivalent tasks (METs), calculated as METs-hours per day by multiplying the reported duration of each activity by its corresponding intensity, as previously described [21]. 

Serum SHBG, E2, TT, and DHEAS levels were quantified using immunoradiometric assay, liquid chromatography-tandem mass spectrometry and radioimmunoassay, respectively. Details of assay sensitivity and variability are available in Supplementary Table 2. Dyslipidemia, hypertension, and diabetes were defined based on established clinical criteria, as detailed in Supplementary Table 3 [22–28]. 

### Statistical analyses

Continuous variables were assessed with the Wilcoxon test, and categorical variables with the chi-squared test. Data are reported as medians (IQR) or frequencies (%).

Logistic regression was employed to evaluate the relationship between the baseline values of four serum hormone measures, SHBG, E2, TT, and DHEAS levels, and the presence of dyslipidemia. To investigate the effect of the 4 serum hormone measures on the presence of dyslipidemia, the following variables were included as independent predictors in the multivariate logistic regression models. In Model 1, age, BMI, physical activity, drinking habits, and smoking status were adjusted for all 570 participants. In Model 2, additional adjustments were made for confounding variables including the presence of hypertension and diabetes along with the variables from Model 1. Model 2 only included 323 participants with complete blood glucose, HbA1c, and glucose-lowering medication data. Associations are expressed as odds ratios (ORs) with corresponding 95% confidence intervals (CIs). Furthermore, for the hormone measure that demonstrated a significant association with dyslipidemia in the multivariate analysis, additional analyses were conducted by categorizing the hormone level into quartiles to evaluate the gradient relationship with the risk of dyslipidemia. A sensitivity analysis was performed, restricted to participants without obesity, defined as BMI < 25 kg/m² [29],.

Furthermore, multiple regression analyses were conducted to evaluate the associations of SHBG, E2, TT, and DHEAS levels with LDL-cholesterol cholesterol, triglyceride, and HDL cholesterol levels. The covariates in Models 1 and 2 were aligned with those used for the logistic regression analysis.

The areas under the curve (AUCs) of SHBG, E2, TT, and DHEAS levels for the presence of dyslipidemia were calculated using a receiver operating characteristic curve (ROC) analysis including sex hormones 1 by 1, and cut-off values for the presence of dyslipidemia were determined. Additionally, the AUCs of SHBG, E2, TT, and DHEAS levels were compared using the DeLong method [30]. Statistical significance was set at a *P* value less than 0.05.

## Results

**Fig. 1:**
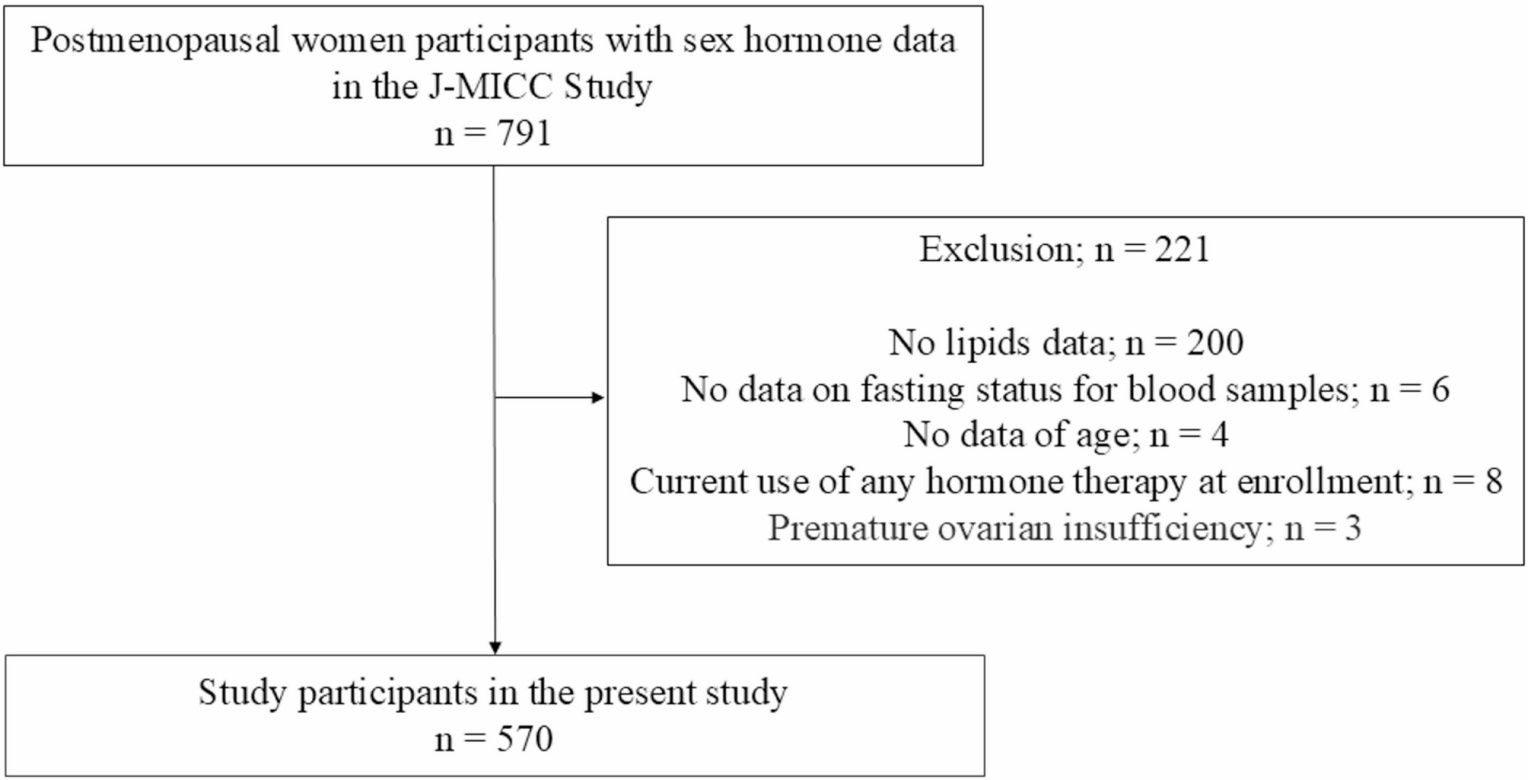
A flow diagram of the participants selection

Among the 791 individuals initially eligible, 221 were excluded due to missing baseline data, resulting in a final analytical cohort of 570 participants. A flowchart outlining the selection process is provided in Fig. 1. Additional analyses were performed after excluding 247 participants without data for blood glucose or HbA1c levels, thus 323 participants remained. Table 1 presents the baseline characteristics of the study cohort. The participants’ median age was 51.0 years (49.0–53.0). Additionally, the median (interquartile range) lipid parameters were as follows: total cholesterol, 221.0 mg/dL (199.0–242.0); triglycerides, 94.0 mg/dL (71.0–132.3); HDL-cholesterol, 65.5 mg/dL (56.7–76.6); and LDL-cholesterol, 129.6 mg/dL (111.6–150.9). At baseline, individuals with dyslipidemia exhibited significantly lower SHBG levels compared to those without dyslipidemia (*P* < 0.001), with median values of 65.2 nmol/L (48.3–86.4) and 80.6 nmol/L (60.4–99.1), respectively. In contrast, no significant differences were observed in E2, TT, or DHEAS levels between individuals with and without dyslipidemia. Median E2 levels were 2.5 pg/mL (1.1–4.1) in individuals with dyslipidemia and 2.6 pg/mL (1.3–4.1) in those without (*P* = 0.370); TT levels were 106.7 pg/mL (80.8–144.4) vs. 110.5 pg/mL (81.8–145.8) (*P* = 0.394); and DHEAS levels were 751.5 ng/mL (509.5–1096.8) vs. 800.5 ng/mL (553.8–1184.3) (*P* = 0.113).

**Table 1:** Characteristics of the participants

	ALL	Dyslipidemia (+)	Dyslipidemia (-)	*P* value
*n*	570	328	242	
Age (years)	51.0 (49.0–53.0)	51.0 (49.3–53.0)	50.0 (48.0–53.0)	0.097
Body Weight (kg)	52.5 (47.8–57.3)	53.8 (49.1–58.7)	51.0 (46.2–55.4)	< 0.001
Body Mass Index (kg/m^2^)	22.6 (20.9–24.8)	23.3 (21.4–25.3)	22.0 (20.1–24.1)	< 0.001
Systolic blood pressure (mmHg)	129.0 (115.0-140.0)	130.0 (117.3–142.0)	126.0 (112.0-139.3)	0.020
Diastolic blood pressure (mmHg)	77.0 (70.0–84.0)	78.0 (70.0–84.0)	76.0 (69.0–82.0)	0.011
Plasma glucose (mg/dL)	93.0 (88.0-100.0)	93.0 (89.0-101.0)	93.0 (88.0–99.0)	0.140
HbA1c (%)	5.6 (5.4–5.8)	5.6 (5.4-6.0)	5.5 (5.4–5.7)	0.001
Uric acid (mg/dL)	4.5 (3.8–5.2)	4.6 (3.9–5.3)	4.4 (3.8–4.9)	0.004
SHBG (nmol/L)	71.1 (51.9–93.0)	65.2 (48.3–86.4)	80.6 (60.4–99.1)	< 0.001
E2 (pg/mL)	2.6 (1.2–4.1)	2.5 (1.1–4.1)	2.6 (1.3–4.1)	0.370
TT (pg/mL)	108.1 (80.8-144.9)	106.7 (80.8-144.4)	110.5 (81.8-145.8)	0.394
DHEAS (ng/mL)	769.5 (525.8-1122.8)	751.5 (509.5-1096.8)	800.5 (553.8-1184.3)	0.113
Smoking status (never/past/current) (%)	537/11/22 (94.2/1.9/3.9)	306/6/16 (93.3/1.8/4.9)	231/5/6 (95.5/2.1/2.5)	0.335
Drinking habits (-/+) (%)	558/12 (97.9/2.1)	321/7 (97.9/2.1)	237/5 (97.9/2.1)	0.958
Physical Activity (MET-hours/day)	26.1 (19.0-36.3)	26.3 (19.0-35.3)	25.7 (19.1–37.5)	0.687

Data were presented as n (%), median (25th, 75th)

Plasma glucose levels and HbA1c levels was obtained from 323 participants out of the total cohort

Abbreviations: SHBG, sex hormone-binding globulin; E2, estradiol; TT, testosterone; DHEAS, dehydroepiandrosterone sulfate

The ORs for the univariate and multivariate logistic regression models for the presence of dyslipidemia are presented in Table 2. Multivariate logistic regression analysis in Model 2 demonstrated a significant association between SHBG levels and the presence of dyslipidemia (OR: 0.745, 95% CI: [0.576–0.958], *P* = 0.022). Similarly, in Model 1, SHBG levels were significantly associated with dyslipidemia, aligning with the findings in Model 2 (OR: 0.728, 95% CI: [0.598–0.884], *P* = 0.001). In addition, multivariable logistic regression analyses were conducted after excluding patients undergoing treatment for dyslipidemia (Supplementary Table 4). In Model 1, SHBG levels were significantly associated with the presence of dyslipidemia (ORs: 0.757, 95% CIs: [0.610–0.934], *P* = 0.010), whereas in Model 2, this association was not significant (ORs: 0.786, 95% CIs: [0.591–1.036], *P* = 0.092). In the sensitivity analysis, a significant association between the SHBG levels and dyslipidemia was also observed among participants without obesity (ORs: 0.688, 95% CIs: [0.523–0.896], *P* = 0.005), as shown in Supplementary Table 5.

**Table 2:** Unadjusted odds ratios and multivariate adjusted odds ratios for dyslipidemia (per standard deviation)

	SHBG	*P*	E2	*P*	TT	*P*	DHEAS	*P*
Crude	0.643 (0.537–0.766)	< 0.001	0.890 (0.734–1.064)	0.220	0.917 (0.744–1.084)	0.311	0.836 (0.707–0.987)	0.035
Model 1	0.728 (0.598–0.884)	0.001	0.881 (0.707–1.047)	0.156	0.938 (0.752–1.113)	0.474	0.850 (0.715–1.007)	0.062
Model 2	0.745 (0.576–0.958)	0.022	0.877 (0.635–1.105)	0.277	0.909 (0.664–1.143)	0.424	0.799 (0.628–1.010)	0.060

Data are presented as odds ratios (95% confidence intervals)

Model 1 was adjusted for age, BMI, physical activity, drinking habits and smoking status with all of 570 participants

Model 2 was adjusted for age, BMI, physical activity, drinking habits, smoking status, hypertension, and diabetes with 323 participants of total cohort

Abbreviations: BMI, body mass index; SHBG, sex hormone-binding globulin; E2, estradiol; TT, total testosterone; DHEAS, dehydroepiandrosterone sulfate

Furthermore, in the multiple regression analyses that evaluated the relationships between SHBG, E2, TT, and DHEAS levels and LDL-cholesterol, triglyceride, and HDL-cholesterol levels, SHBG levels had a significant negative correlation with triglyceride levels (β = -0.217, *P* < 0.001) and a significant positive correlation with HDL cholesterol levels (β = 0.136, *P* = 0.016), whereas no significant association was observed with LDL-cholesterol levels (β = -0.062, *P* = 0.296), as shown in Supplementary Table 6. Among the sex hormones except SHBG levels, E2 levels were positively associated with HDL cholesterol levels (β = 0.131, *P* = 0.012). In addition, an exploratory analysis was conducted to examine the association between SHBG quartiles and dyslipidemia risk. Supplementary Table 7 presents that higher SHBG levels were associated with lower odds of dyslipidemia, displaying a significant inverse trend observed across quartiles (*P* for trend = 0.046), thereby suggesting a potential graded relationship.

**Fig. 2:**
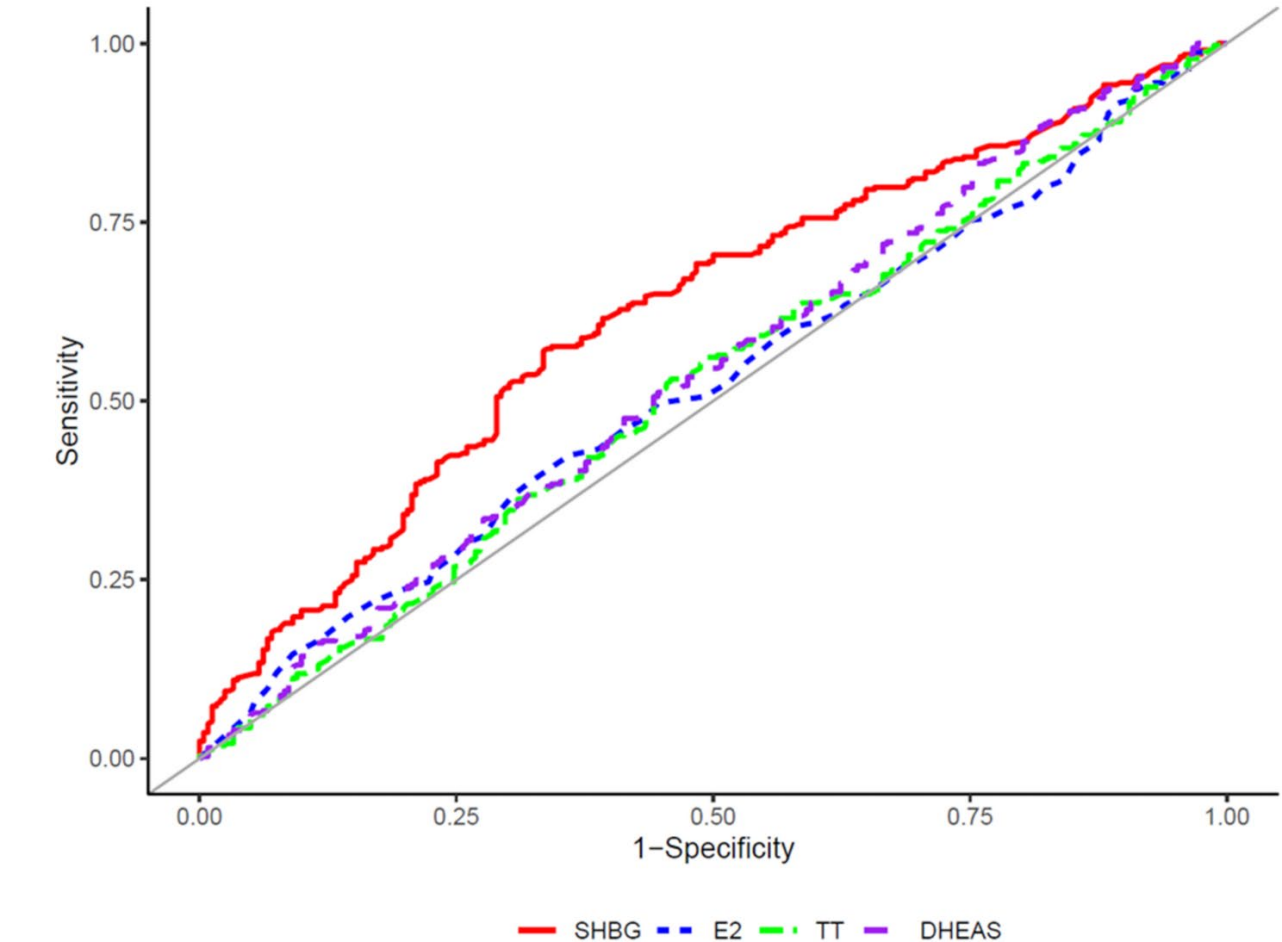
Area under the receiver operating characteristic curve showing the ability of SHBG, E2, TT, DHEAS for the presence of dyslipidemia. Abbreviations: SHBG, sex hormone-binding globulin; E2, estradiol; TT, Total testosterone; DHEAS, dehydroepiandrosterone-sulfate

The AUC and cutoff values for the presence of dyslipidemia were presented using an ROC curve analysis (Table 3). The AUC and optimal cutoff values for SHBG levels were 0.626 and 69.0 nmol/L, respectively. Figure 2 shows the ROC curves demonstrating the associations of SHBG, E2, TT, and DHEAS levels with dyslipidemia. Table 4 lists the results for the comparison of AUCs for SHBG, E2, TT, and DHEAS levels. The AUC for SHBG was significantly higher than those for E2, TT, and DHEAS, with *P* values of 0.005, 0.001, and 0.022, respectively.

**Table 3:** Area under the curve and optimal cut-off values

	AUC (95% CI)	Sensitivity	Specificity	Cut-off value
SHBG	0.626 (0.580–0.672)	57.0%	66.5%	69.0
E2	0.522 (0.474–0.570)	38.7%	67.8%	1.85
TT	0.521 (0.473–0.569)	53.0%	54.1%	108.1
DHEAS	0.539 (0.491–0.587)	83.2%	24.4%	1217.0

Abbreviations: SHBG, sex hormone-binding globulin; E2, estradiol; TT, total testosterone; DHEAS, dehydroepiandrosterone sulfate

**Table 4:** Comparison of area under the curve for sex hormones with the presence of dyslipidemia

	vs. SHBG		vs. E2		vs. TT		vs. DHEAS	
	difference value	95% CI *P* value	difference value	95% CI *P* value	difference value	95% CI *P* value	difference value	95% CI *P* value
SHBG	reference		-		-		-	
E2	-0.104	-0.176 to -0.032 *P* = 0.005	reference		-		-	
TT	-0.105	-0.168 to -0.041 *P* = 0.001	-0.001	-0.060 to 0.058 *P* = 0.972	reference		-	
DHEAS	-0.087	-0.161 to -0.013 *P* = 0.022	0.017	-0.043 to 0.077 *P* = 0.580	0.018	-0.038 to 0.074 *P* = 0.529	reference	

Abbreviations: SHBG, sex hormone-binding globulin; E2, estradiol; TT, total testosterone; DHEAS, dehydroepiandrosterone sulfate

## Discussion

The main study findings for postmenopausal women participants were: (1) SHBG levels were significantly associated with dyslipidemia, (2) SHBG levels were positively correlated with HDL cholesterol levels and inversely correlated with triglyceride levels, and (3) the AUC for SHBG levels was the highest among those for E2, TT, and DHEAS levels. The optimal cutoff value for SHBG levels in identifying dyslipidemia was determined to be 69.0 nmol/L.

Although the primary role of circulating SHBG is to bind sex hormones and regulate their bioavailability, recent studies have demonstrated that SHBG also plays an active role in androgen signaling and other functions [31]. These findings highlight the multifaceted nature of SHBG and its broad implications in metabolic and hormonal regulation.

Over the past few decades, low SHBG levels have consistently been linked to an increased risk of developing various metabolic disorders [8, 9, 11]. and in this study, SHBG was significantly correlated with dyslipidemia. Importantly, reduced SHBG levels have been linked to insulin resistance and a heightened risk of developing type 2 diabetes mellitus, irrespective of other sex hormones, in both sexes [10]. Strain et al. [32] have previously documented that insulin inhibits SHBG synthesis, suggesting that hyperinsulinemia resulting from insulin resistance contributes to reduced SHBG levels. Insulin resistance is often accompanied by hypertriglyceridemia and reduced HDL cholesterol levels [33], with the former primarily attributed to the overproduction of very low-density lipoproteins [33], whereas the latter may result from increased hepatic triglyceride lipase and accelerated clearance of HDL cholesterol [34]. In the present study, low SHBG levels were associated with hypertriglyceridemia and low HDL cholesterol levels in postmenopausal women, which is consistent with previous research [11]. These findings further support the link between SHBG levels and insulin resistance.

Khoudary et al. [35] revealed that E2 levels are associated with HDL cholesterol function during menopausal transition. In line with this finding, the present study demonstrated a positive correlation between E2 and HDL cholesterol levels in postmenopausal women. Although low HDL cholesterol levels are known to be associated with an increased risk of coronary artery disease [36], evidence also links very high HDL cholesterol levels to adverse cardiovascular outcomes [37]. Therefore, further research is necessary to establish optimal management strategies for estradiol deficiency in postmenopausal women including E2 hormone therapy and target-setting approaches.

Although the association between E2 and SHBG levels and dyslipidemia in postmenopausal women has been consistently reported, the relationship between testosterone and dyslipidemia in these women remains unclear because evidence both supports and opposes [13, 38, 39]. In this study, no significant association between testosterone levels and dyslipidemia was observed. Previous studies have demonstrated a significant relationship between testosterone levels and dyslipidemia, including in participants with a higher average age than those in the present study [38, 39]. Triglyceride levels are the highest between 10 and 14 years after menopause [40] which suggests that age might be a contributing factor to these conflicting findings.

In this study, the association between SHBG and dyslipidemia differed from that observed in the case of E2 and TT. SHBG functions as an estradiol and testosterone binding protein, regulating various biological processes [10]. In postmenopausal women, biologically active free hormone levels are substantially reduced [41, 42]. Therefore, the observed relationship between SHBG and dyslipidemia might reflect sex steroid activity-independent mechanisms, suggesting the potentially direct role of SHBG in lipid metabolism.

Noyan et al. [13] reported a positive correlation between DHEAS and HDL-cholesterol levels. Although the association between DHEAS, HDL cholesterol levels and dyslipidemia was not statistically significant in this study, there were trends suggesting a relationship.

The present findings indicated that SHBG levels could be the most reliable indicator of dyslipidemia among E2, TT, and DHEAS levels, as suggested by the AUC comparisons. Thus, these findings suggest the importance of SHBG levels in relation to dyslipidemia. Although this study demonstrated the potential utility of SHBG in identifying dyslipidemia, its discriminatory ability alone, as reflected by the AUC, was suboptimal. Accordingly, SHBG should be considered as part of a broader risk assessment framework, in conjunction with other clinical parameters, to enhance diagnostic accuracy.

### Strengths and limitations

The strength of this study is that it is the first to comprehensively compare the associations of dyslipidemia with SHBG, E2, TT, and DHEAS. Several limitations should be acknowledged. Primarily, its cross-sectional design restricts the interpretation of any causal link between circulating sex hormones and dyslipidemia. Second, this study population was limited to Japanese individuals. Consequently, the potential for selection bias cannot be excluded, which limits the generalizability of the findings to other ethnic groups. Third, smoking status and alcohol consumption were self-reported, which may have introduced inaccuracies. Nonetheless, previous studies have indicated that self-reported data can be useful for assessing smoking status and drinking behaviors [43, 44], and the lifestyle data collected in the present study were considered valid. Fourth, the present study lacks data on several covariates that could potentially influence dyslipidemia, such as dietary patterns [45], thyroid hormones [46], visceral fat indicator (e.g., waist circumference), and an insulin resistance marker (e.g., insulin resistance homeostasis model assessment). Moreover, both thyroid function and insulin resistance reportedly affect SHBG levels [10, 47]. Therefore, the absence of relevant markers, including thyroid hormones and surrogate insulin resistance markers, represents an additional limitation. Finally, data on FSH, a key hormone for characterizing the hormonal status in postmenopausal women, were not available in this study. However, as FSH is significantly positively correlated with SHBG [7], such absence is unlikely to substantially compromise the clinical relevance of the present results.

## Conclusions

In conclusion, among postmenopausal women, SHBG levels were most strongly associated with dyslipidemia among E2, TT, and DHEAS levels. This study also determined that the optimal SHBG cutoff value for the presence of dyslipidemia was 69.0 nmol/L. These findings highlight SHBG as a promising biomarker for identifying postmenopausal women at increased risk of dyslipidemia. Future studies should focus on monitoring postmenopausal women with SHBG levels above this threshold for the development of dyslipidemia.

## Electronic supplementary material

Below is the link to the electronic supplementary material.

Supplementary Material 1

Supplementary Material 2

Supplementary Material 3

Supplementary Material 4

Supplementary Material 5

Supplementary Material 6

Supplementary Material 7

## Data Availability

Data are available upon reasonable request. Details can be found on the J-MICC Study website (http://www.jmicc.com/).

## Abbreviations

AUCs: Areas under the curve
BMI: Body mass index
CIs: Confidence intervals
DHEAS: Dehydroepiandrosterone sulfate
E2: Estradiol
FSH: Follicle-stimulating hormone
HDL: Cholesterol high density lipoprotein cholesterol
IRMA: Immunoradiometric assay
LC-MS/MS: Liquid chromatography-tandem mass spectrometry
LDL-cholesterol: Low density lipoprotein cholesterol
METs: Metabolic equivalents
ORs: Odds ratios
RIA: Radioimmunoassay
ROC: Receiver operating characteristic curve
SHBG: Sex hormone-binding globulin
TT: Testosterone

## References

[1] Pirillo A, Casula M, Olmastroni E, Norata GD, Catapano AL. Global epidemiology of dyslipidaemias. Nat Rev Cardiol. 2021;18:689–700. 33833450 10.1038/s41569-021-00541-4

[2] Yusuf S, Hawken S, Ôunpuu S, et al. Effect of potentially modifiable risk factors associated with myocardial infarction in 52 countries (the INTERHEART study): case-control study. Lancet. 2004;364:937–52. 15364185 10.1016/S0140-6736(04)17018-9

[3] El Khoudary SR, Aggarwal B, Beckie TM, Hodis HN, Johnson AE, Langer RD, Limacher MC, Manson JE, Stefanick ML, Allison MA. Menopause transition and cardiovascular disease risk: implications for timing of early prevention: a scientific statement from the American Heart Association. Circulation. 2020;142:E506–32. 33251828 10.1161/CIR.0000000000000912

[4] Lamberts SWJ, van den Beld AW, van der Lely A-J. The endocrinology of aging. Sci (1979). 1997;278:419–24. 10.1126/science.278.5337.419 9334293

[5] Pasquali R, Vicennati V, Bertazzo D, Casimirri F, Pascal G, Tortelli O, Labate AMM. Determinants of sex hormone—binding globulin blood concentrations in premenopausal and postmenopausal women with different estrogen status. Metabolism. 1997;46:5–9. 9005961 10.1016/s0026-0495(97)90159-1

[6] Burger HG. The endocrinology of the menopause. Maturitas. 1996;23:129–36. 8735351 10.1016/0378-5122(95)00969-8

[7] Wang N, Zhang K, Han B, et al. Follicle stimulating hormone, its novel association with sex hormone binding globulin in men and postmenopausal women. Endocrine. 2017;56:649–57. 28260206 10.1007/s12020-017-1272-y

[8] Ding EL, Song Y, Manson JE, Hunter DJ, Lee CC, Rifai N, Buring JE, Gaziano JM, Liu S. Sex hormone–binding globulin and risk of type 2 diabetes in women and men. N Engl J Med. 2009;361:1152–63. 19657112 10.1056/NEJMoa0804381 PMC2774225

[9] Watz MES, Tivesten A, Ottarsdottir K, Li Y, Hellgren MI, Lindblad U, Daka B. Sex hormone-binding globulin levels and development of hypertension in middle-aged men and women. J Hypertens. 2023;41:1565–70. 37436403 10.1097/HJH.0000000000003506

[10] Wallace IR, McKinley MC, Bell PM, Hunter SJ. Sex hormone binding globulin and insulin resistance. Clin Endocrinol (Oxf). 2013;78:321–9. 23121642 10.1111/cen.12086

[11] Haffner SM, Dunn JF, Katz MS. Relationship of sex hormone-binding globulin to lipid, lipoprotein, glucose, and insulin concentrations in postmenopausal women. Metabolism. 1992;41:278–84. 1542267 10.1016/0026-0495(92)90271-b

[12] Rexrode KM, Manson JAE, Lee IM, Ridker PM, Sluss PM, Cook NR, Buring JE. Sex hormone levels and risk of cardiovascular events in postmenopausal women. Circulation. 2003;108:1688–93. 12975257 10.1161/01.CIR.0000091114.36254.F3

[13] Noyan V, Yucel A, Sagsoz N. The association of androgenic sex steroids with serum lipid levels in postmenopausal women. Acta Obstet Gynecol Scand. 2004;83:487–90. 15059164 10.1111/j.0001-6349.2004.00417.x

[14] Kumagai S, Kai Y, Sasaki H. Relationship between insulin resistance, sex hormones and sex hormone-binding globulin in the serum lipid and lipoprotein profiles of Japanese postmenopausal women. J Atheroscler Thromb. 2001;8:14–20. 11686310 10.5551/jat1994.8.14

[15] Mudali S, Dobs AS, Ding J, Cauley JA, Szklo M, Golden SH. Endogenous postmenopausal hormones and serum lipids: the atherosclerosis risk in communities study. J Clin Endocrinol Metab. 2005;90:1202–9. 15546905 10.1210/jc.2004-0744

[16] Takeuchi K, Naito M, Kawai S, et al. Study profile of the Japan multi-institutional collaborative cohort (J-MICC) study. J Epidemiol. 2021;31:JE20200147. 10.2188/jea.JE20200147 PMC8593573 32963210

[17] Wakai K, Hamajima N, Okada R, et al. Profile of participants and genotype distributions of 108 polymorphisms in a cross-sectional study of associations of genotypes with lifestyle and clinical factors: a project in the Japan Multi-Institutional Collaborative Cohort (J-MICC) study. J Epidemiol. 2011;21:223–35. 21467728 10.2188/jea.JE20100139 PMC3899413

[18] Uemura H, Hiyoshi M, Arisawa K, et al. Gene variants in PPARD and PPARGC1A are associated with timing of natural menopause in the general Japanese population. Maturitas. 2012;71:369–75. 22310107 10.1016/j.maturitas.2011.12.021

[19] Zhang X, Liu Y, Li S, et al. Alcohol consumption and risk of cardiovascular disease, cancer and mortality: a prospective cohort study. Nutr J. 2021. 10.1186/s12937-021-00671-y 33522924 10.1186/s12937-021-00671-y PMC7852289

[20] Craig CL, Marshall AL, Sjöström M, et al. International physical activity questionnaire: 12-country reliability and validity. Med Sci Sports Exerc. 2003;35:1381–95. 12900694 10.1249/01.MSS.0000078924.61453.FB

[21] Hara M, Higaki Y, Taguchi N, et al. Effect of the PPARG2 Pro12Ala polymorphism and clinical risk factors for diabetes mellitus on HbA1c in the Japanese general population. J Epidemiol. 2012;22:523–31. 23006958 10.2188/jea.JE20120078 PMC3798564

[22] Teramoto T, Sasaki J, Birou S et al. Diagnostic criteria for dyslipidemia executive summary of the Japan Atherosclerosis Society (JAS) Guidelines for the diagnosis and prevention of atherosclerotic cardiovascular diseases in Japan-2012 Version. 2013. 10.5551/jat.17368 23892529

[23] Nordestgaard BG, Langsted A, Mora S, et al. Fasting is not routinely required for determination of a lipid profile: clinical and laboratory implications including flagging at desirable concentration cut-points - a joint consensus statement from the European Atherosclerosis Society and European Federation of Clinical Chemistry and Laboratory Medicine. Eur Heart J. 2016;37:1944–58. 27122601 10.1093/eurheartj/ehw152 PMC4929379

[24] Doran B, Guo Y, Xu J, Weintraub H, Mora S, Maron DJ, Bangalore S. Prognostic value of fasting versus nonfasting low-density lipoprotein cholesterol levels on long-term mortality: insight from the National Health and Nutrition Examination Survey III (NHANES-III). Circulation. 2014;130:546–53. 25015340 10.1161/CIRCULATIONAHA.114.010001

[25] Nordestgaard BG. A test in context: lipid profile, fasting versus nonfasting. J Am Coll Cardiol. 2017;70:1637–46. 28935041 10.1016/j.jacc.2017.08.006

[26] Unger T, Borghi C, Charchar F, et al. 2020 international society of hypertension global hypertension practice guidelines. Hypertension. 2020;75:1334–57. 32370572 10.1161/HYPERTENSIONAHA.120.15026

[27] Araki E, Goto A, Kondo T, et al. Japanese clinical practice guideline for diabetes 2019. Diabetol Int. 2020;11:165–223. 32802702 10.1007/s13340-020-00439-5 PMC7387396

[28] ElSayed NA, Aleppo G, Bannuru RR, et al. 2. Diagnosis and classification of diabetes: *Standards of care in Diabetes—2024*. Diabetes Care. 2024;47:S20–42. 38078589 10.2337/dc24-S002 PMC10725812

[29] Goda A, Masuyama T. Obesity and overweight in Asian people. Circ J. 2016;80:2425–6. 27829594 10.1253/circj.CJ-16-1087

[30] DeLong ER, DeLong DM, Clarke-Pearson DL. Comparing the areas under two or more correlated receiver operating characteristic curves: a nonparametric approach. Biometrics. 1988;44:837. 3203132

[31] Basualto-Alarcón C, Llanos P, García-Rivas G, Troncoso MF, Lagos D, Barrientos G, Estrada M. Classic and novel sex hormone binding globulin effects on the cardiovascular system in men. Int J Endocrinol. 2021. 10.1155/2021/5527973 34335746 10.1155/2021/5527973 PMC8318754

[32] Strain G, Zumoff B, Rosner W, Pi-Sunyer X. The relationship between serum levels of insulin and sex hormone-binding globulin in men: the effect of weight loss. J Clin Endocrinol Metab. 1994;79:1173–6. 7962291 10.1210/jcem.79.4.7962291

[33] Rashid S, Uffelman KD, Lewis GF. The mechanism of HDL lowering in hypertriglyceridemic, insulin-resistant states. J Diabetes Complications. 2002;16:24–8. 11872362 10.1016/s1056-8727(01)00191-x

[34] Bjornstad P, Eckel RH. Pathogenesis of lipid disorders in insulin resistance: a brief review. Curr Diab Rep. 2018. 10.1007/s11892-018-1101-6 30328521 10.1007/s11892-018-1101-6 PMC6428207

[35] El Khoudary SR, Nasr A, Billheimer J, Brooks MM, McConnell D, Crawford S, Orchard TJ, Rader DJ, Matthews KA. Associations of endogenous hormones with HDL novel metrics across the menopause transition: the SWAN HDL study. J Clin Endocrinol Metab. 2022;107:E303–14. 34390340 10.1210/clinem/dgab595 PMC8684446

[36] Toth PP. High-density lipoprotein and cardiovascular risk. Circulation. 2004;109:1809–12. 15096460 10.1161/01.CIR.0000126889.97626.B8

[37] Liu C, Dhindsa D, Almuwaqqat Z, et al. Association between high-density lipoprotein cholesterol levels and adverse cardiovascular outcomes in high-risk populations. JAMA Cardiol. 2022;7:672. 35583863 10.1001/jamacardio.2022.0912 PMC9118072

[38] Kumagai S, Kai Y, Sasaki H. Relationship between insulin resistance, sex hormones and sex hormone-binding globulin in the serum lipid and lipoprotein profiles of Japanese postmenopausal women. 2001. 10.5551/jat1994.8.14 11686310

[39] Davis SR, Azene ZN, Tonkin AM, Woods RL, McNeil JJ, Islam RM. Higher testosterone is associated with higher HDL-cholesterol and lower triglyceride concentrations in older women: an observational study. Climacteric. 2024;27:282–8. 38345304 10.1080/13697137.2024.2310530 PMC11196127

[40] Joon Cho G, Hyun Lee J, Tae Park H, Ho Shin J, Cheol Hong S, Kim T, Young Hur J, Wan Lee K, Kyun Park Y, Haeng Kim S. Postmenopausal status according to years since menopause as an independent risk factor for the metabolic syndrome. Menopause. 2008;15:524–9. 18467953 10.1097/gme.0b013e3181559860

[41] Burger HG. Hormonal changes in the menopause transition. Recent Prog Horm Res. 2002;57:257–75. 12017547 10.1210/rp.57.1.257

[42] Longcope C, Hui SL, Johnston CC. Free estradiol, free testosterone, and sex hormone-binding globulin in perimenopausal women**. J Clin Endocrinol Metab. 1987;64:513–8. 3102538 10.1210/jcem-64-3-513

[43] Wong SL, Shields M, Leatherdale S, Malaison E, Hammond D. Assessment of validity of self-reported smoking status. Health Rep. 2012;23:47–53. 22590805

[44] Del Boca FK, Darkes J. The validity of self-reports of alcohol consumption: state of the science and challenges for research. Addiction. 2003;98:1–12. 14984237 10.1046/j.1359-6357.2003.00586.x

[45] Agarwala A, Petersen KS, Jafari F, Kris-Etherton PM. Dietary management of dyslipidemia and the impact of dietary patterns on lipid disorders. Prog Cardiovasc Dis. 2022;75:49–58. 36410416 10.1016/j.pcad.2022.11.003

[46] Duntas LH. Thyroid disease and lipids. Thyroid. 2002;12:287–93. 12034052 10.1089/10507250252949405

[47] Selva DM, Hammond GL. Thyroid hormones act indirectly to increase sex hormone-binding globulin production by liver via hepatocyte nuclear factor-4α. J Mol Endocrinol. 2009;43:19–27. 19336534 10.1677/JME-09-0025

