# Supplementary material for: Association of sex hormone-binding globulin and dyslipidemia with Japanese postmenopausal women: a cross-sectional study
Source: Lipids Health Dis. 2025 Jun 10;24:212. doi: 10.1186/s12944-025-02634-2 (PMC12150563; doi:10.1186/s12944-025-02634-2)
Supplement: Supplementary file 1 — Supplementary Material 1 [file 12944_2025_2634_MOESM1_ESM.docx]

Supplementary Table 1. Details of the Japan Multi-Institutional Collaborative Cohort Study.

A total of more than 90,000 participants from 14 regions across Japan were initially enrolled in the baseline survey. To investigate the association between lifestyle, genetic, and medical factors, a subset of 500-600 consecutive subjects was selected from ten regions, ultimately resulting in 4,519 participants (2,124 men and 2,395 women) for the cross-sectional study. This study was approved by the Institutional Ethics Committee of Kyoto Prefectural University of Medicine (approval number: ERB-C-3202 at 2024) and was conducted in accordance with the principles outlined in the Declaration of Helsinki. Additionally, all procedures involving human subjects were approved by the ethics committee of Nagoya University Graduate School of Medicine, Aichi Cancer Center, and the respective ethics committees of all participating institutions.
