# Supplementary material for: Association of sex hormone-binding globulin and dyslipidemia with Japanese postmenopausal women: a cross-sectional study
Source: Lipids Health Dis. 2025 Jun 10;24:212. doi: 10.1186/s12944-025-02634-2 (PMC12150563; doi:10.1186/s12944-025-02634-2)
Supplement: Supplementary file 2 — Supplementary Material 2 [file 12944_2025_2634_MOESM2_ESM.docx]

Supplementary Table 2. Serum sample analysis.

Serum samples were frozen and kept at -80℃ until the measurement. Serum SHBG levels were measured using an immunoradiometric assay (IRMA) and serum E2 and TT levels were measured using liquid chromatography-tandem mass spectrometry (LC-MS/MS). DHEAS was measured using a radioimmunoassay (RIA). The analyses were conducted at ASKA Pharmaceutical Co., Ltd. The sensitivity of LC-MS/MS were 5.0 pg/ml for E2, 10.0 pg/mL for TT. In addition, the sensitivity of IRMA was 0.2 nmol/l for SHBG and that of RIA was 50.0 ng/mL for DHEAS. The intra assay CV were 3.2-6.6% for E2, and 1.4-2.8% for TT, 3.5-6.5% for SHBG. The inter-assay CV for E2, TT, and SHBG were 4.8-9.5, 3.4-5.1, 1.5-8.2%, respectively.
