# Supplementary material for: Association of sex hormone-binding globulin and dyslipidemia with Japanese postmenopausal women: a cross-sectional study
Source: Lipids Health Dis. 2025 Jun 10;24:212. doi: 10.1186/s12944-025-02634-2 (PMC12150563; doi:10.1186/s12944-025-02634-2)
Supplement: Supplementary file 3 — Supplementary Material 3 [file 12944_2025_2634_MOESM3_ESM.docx]

Supplementary Table 3. Definition of dyslipidemia, hypertension, and diabetes

Dyslipidemia was defined as either LDL-cholesterol levels of ≥140 mg/dL, HDL-cholesterol levels of ≤40 mg/dL, and triglyceride levels ≥150 mg/dL, or as the current use of lipid-lowering medications. In non-fasting conditions, dyslipidemia was defined as triglycerides levels ≥175 mg/dL, HDL-cholesterol level of ≤40 mg/dL, and LDL-cholesterol levels was considered equivalent to fasting LDL-cholesterol levels of ≥140 mg/dL. Fasting time was defined as a period of ≥8 hours. Hypertension was defined as a systolic blood pressure of ≥140 mmHg, a diastolic blood pressure of ≥90 mmHg, or the current use of antihypertension medications. Diabetes was diagnosed by fasting glucose concentrations ≥126 mg/dL or random glucose ≥200 mg/dL, in conjunction with HbA1c ≥6.5%. Fasting, defined as no caloric intake for a minimum of 8 hours, was required for diagnosing diabetes. In addition, current use of glucose-lowering medications was defined as diabetes.
