# Supplementary material for: Association of sex hormone-binding globulin and dyslipidemia with Japanese postmenopausal women: a cross-sectional study
Source: Lipids Health Dis. 2025 Jun 10;24:212. doi: 10.1186/s12944-025-02634-2 (PMC12150563; doi:10.1186/s12944-025-02634-2)
Supplement: Supplementary file 4 — Supplementary Material 4 [file 12944_2025_2634_MOESM4_ESM.docx]

Supplementary Table 4. Unadjusted odds ratios and multivariate adjusted odds ratios for dyslipidemia among participants not using lipid-lowering medications. (per standard deviation)

|  | SHBG | *P* | E2 | *P* | TT | *P* | DHEAS | *P* |
| --- | --- | --- | --- | --- | --- | --- | --- | --- |
| Crude | 0.686 (0.565-0.828) | <0.001 | 0.924 (0.750-1.094) | 0.386 | 0.945 (0.768-1.117) | 0.520 | 0.898 (0.753-1.068) | 0.227 |
| Model 1 | 0.757 (0.610-0.934) | 0.010 | 0.906 (0.724-1.079) | 0.309 | 0.963 (0.776-1.141) | 0.678 | 0.906 (0.756-1.082) | 0.276 |
| Model 2 | 0.786 (0.591-1.036) | 0.092 | 0.895 (0.638-1.132) | 0.374 | 0.943 (0.694-1.185) | 0.626 | 0.845 (0.656-1.080) | 0.180 |

Data are presented as odds ratios (95% confidence intervals).

Model 1 was adjusted for age, BMI, physical activity, drinking habits, and smoking status in a cohort of 481 participants who were not using lipid-lowering medications.

Model 2 was adjusted for age, BMI, physical activity, drinking habits, smoking status, hypertension, and diabetes in a cohort of 262 participants who were not using lipid-lowering medications.

Abbreviations: BMI, body mass index; SHBG, sex hormone-binding globulin; E2, estradiol; TT, total testosterone; DHEAS, dehydroepiandrosterone sulfate.
