# Supplementary material for: Association of sex hormone-binding globulin and dyslipidemia with Japanese postmenopausal women: a cross-sectional study
Source: Lipids Health Dis. 2025 Jun 10;24:212. doi: 10.1186/s12944-025-02634-2 (PMC12150563; doi:10.1186/s12944-025-02634-2)
Supplement: Supplementary file 5 — Supplementary Material 5 [file 12944_2025_2634_MOESM5_ESM.docx]

Supplementary Table 5. Unadjusted odds ratios and multivariate adjusted odds ratios for dyslipidemia among the participants without obesity (per standard deviation)

|  | SHBG | *P* | E2 | *P* | TT | *P* | DHEAS | *P* |
| --- | --- | --- | --- | --- | --- | --- | --- | --- |
| Crude | 0.666 (0.541-0.815) | <0.001 | 0.883 (0.705-1.051) | 0.202 | 0.928 (0.753-1.098) | 0.400 | 0.817 (0.677-0.981) | 0.032 |
| Model 1 | 0.669 (0.542-0.819) | <0.001 | 0.879 (0.696-1.050) | 0.162 | 0.926 (0.752-1.096) | 0.372 | 0.817 (0.676-0.982) | 0.032 |
| Model 2 | 0.688 (0.523-0.896) | 0.005 | 0.880 (0.626-1.115) | 0.307 | 0.879 (0.647-1.101) | 0.266 | 0.810 (0.626-1.042) | 0.101 |

Data are presented as odds ratios (95% confidence intervals).

Model 1 was adjusted for age, physical activity, drinking habits and smoking status with all of 437 participants without obesity.

Model 2 was adjusted for age, physical activity, drinking habits, smoking status, diabetes, and hypertension with 253 participants without obesity of total cohort.

Abbreviations: SHBG, sex hormone-binding globulin; E2, estradiol; TT, total testosterone; DHEAS, dehydroepiandrosterone sulfate.
