# Supplementary material for: Association of sex hormone-binding globulin and dyslipidemia with Japanese postmenopausal women: a cross-sectional study
Source: Lipids Health Dis. 2025 Jun 10;24:212. doi: 10.1186/s12944-025-02634-2 (PMC12150563; doi:10.1186/s12944-025-02634-2)
Supplement: Supplementary file 6 — Supplementary Material 6 [file 12944_2025_2634_MOESM6_ESM.docx]

Supplementary Table 6. Unadjusted and adjusted associations of sex hormones with lipids

| LDL-C | SHBG |  | E2 |  | TT |  | DHEAS |  |
| --- | --- | --- | --- | --- | --- | --- | --- | --- |
|  | β | *P* | β | *P* | β | *P* | β | *P* |
| Crude | -0.045 | 0.282 | -0.026 | 0.534 | -0.024 | 0.565 | 0.007 | 0.874 |
| Model 1 | -0.019 | 0.678 | -0.031 | 0.454 | -0.019 | 0.643 | 0.014 | 0.737 |
| Model 2 | -0.062 | 0.296 | -0.037 | 0.502 | 0.002 | 0.977 | -0.015 | 0.790 |
| TG | SHBG |  | E2 |  | TT |  | DHEAS |  |
|  | β | *P* | β | *P* | β | *P* | β | *P* |
| Crude | -0.306 | <0.001 | -0.037 | 0.384 | -0.052 | 0.215 | -0.037 | 0.374 |
| Model 1 | -0.295 | <0.001 | -0.038 | 0.356 | -0.040 | 0.334 | -0.025 | 0.543 |
| Model 2 | -0.217 | <0.001 | -0.072 | 0.185 | -0.078 | 0.154 | -0.067 | 0.225 |
| HDL-C | SHBG |  | E2 |  | TT |  | DHEAS |  |
|  | β | *P* | β | *P* | β | *P* | β | *P* |
| Crude | 0.258 | <0.001 | 0.083 | 0.047 | 0.083 | 0.048 | 0.121 | 0.004 |
| Model 1 | 0.163 | <0.001 | 0.090 | 0.023 | 0.063 | 0.114 | 0.097 | 0.015 |
| Model 2 | 0.136 | 0.016 | 0.131 | 0.012 | 0.080 | 0.129 | 0.127 | 0.082 |

Model 1 was adjusted for age, BMI, physical activity, drinking habits and smoking status with all of 570 participants.

Model 2 was adjusted for age, BMI, physical activity, drinking habits, smoking status, hypertension, and diabetes with 323 participants of total cohort.

Abbreviations: LDL-C, low-density lipoprotein cholesterol; TG, triglyceride; HDL-C, high-density lipoprotein cholesterol; SHBG, sex hormone-binding globulin; E2, estradiol; TT, total testosterone; DHEAS, dehydroepiandrosterone sulfate.
