# Supplementary material for: Association of sex hormone-binding globulin and dyslipidemia with Japanese postmenopausal women: a cross-sectional study
Source: Lipids Health Dis. 2025 Jun 10;24:212. doi: 10.1186/s12944-025-02634-2 (PMC12150563; doi:10.1186/s12944-025-02634-2)
Supplement: Supplementary file 7 — Supplementary Material 7 [file 12944_2025_2634_MOESM7_ESM.docx]

Supplementary Table 7. Odds ratios for associations between SHBG quartiles and the presence of dyslipidemia (95% CI)

| SHBG  (nmol/L) | Q1  (-56.4) | Q2  (56.4-75.0) | Q3  (56.4-75.0) | Q4  (99.1-) | *P*-values for trend |
| --- | --- | --- | --- | --- | --- |
| Crude | Ref. | 0.91 (0.55-1.49) | 0.43 (0.26-0.69) | 0.39 (0.24-0.63) | <0.001 |
| Model 1 | Ref | 1.01 (0.61-1.68) | 0.54 (0.32-0.90) | 0.55 (0.32-0.93) | 0.005 |
| Model 2 | Ref. | 1.24 (0.62-2.47) | 0.59 (0.30-1.16) | 0.61 (0.30-1.20) | 0.046 |

Data are presented as odds ratios (95% confidence intervals).

Model 1 was adjusted for age, BMI, physical activity, drinking habits and smoking status with all of 570 participants.

Model 2 was adjusted for age, BMI, physical activity, drinking habits, smoking status, hypertension, and diabetes with 323 participants of total cohort.

Abbreviations: BMI, body mass index; SHBG, sex hormone-binding globulin; E2, estradiol; TT, total testosterone; DHEAS, dehydroepiandrosterone sulfate.
